# Effect of an Ultrasound Pre-Treatment on the Characteristics and Quality of Far-Infrared Vacuum Drying with Cistanche Slices

**DOI:** 10.3390/foods11060866

**Published:** 2022-03-18

**Authors:** Chunhui Jiang, Fangxin Wan, Zepeng Zang, Qian Zhang, Guojun Ma, Xiaopeng Huang

**Affiliations:** College of Electrical and Mechanical Engineering, Gansu Agricultural University, Lanzhou 730070, China; jiangchunh@st.gsau.edu.cn (C.J.); zangzp@st.gsau.edu.cn (Z.Z.); zhangq@st.gsau.edu.cn (Q.Z.); magj@gsau.edu.cn (G.M.)

**Keywords:** ultrasound pre-treatment, Cistanche slices, far-infrared vacuum drying, drying characteristics, drying quality

## Abstract

In this study, the effect of an ultrasound (US) pre-treatment on the process of drying Cistanche slices through far-infrared vacuum drying was investigated with various experimental factors, including the US treatment time (25, 35, 45 min), frequency (20, 40, 60 kHz) and power (150, 180, 210 W). The results showed that compared with the samples without US, the material drying time after the US treatment was reduced by 16–36.8%. The effective moisture diffusion coefficients of Cistanche slices under different US conditions ranged from 1.61122 × 10^−8^ to 2.39274 × 10^−8^ m^2^/s, which agreed with food processing ranges. In addition, the phenylethanoid glycoside, iridoid, polysaccharide, total phenol and total flavonoid contents in Cistanche were significantly increased after US pre-treatment. However, the dried products obtained with the 45 min US treatment had greatly damaged internal structures, collapsed and seriously deformed surfaces, and low contents of active ingredients. Overall, the US pre-treatment could significantly improve the drying quality of Cistanche slices.

## 1. Introduction

Cistanche deserticola Ma, or Da Yun, is a member of the genus Cistanche. It is mainly distributed in arid and semi-arid environments in China, Iran, Mongolia and other parts of Eurasia and North Africa [1]. Cistanche has the function of moistening intestines and relaxing bowels, tonifying kidney and replenishing essence. The frequency of Cistanche in Chinese supplementary prescriptions is second only to ginseng and has the reputation of “desert ginseng” [2]. It is now often processed as capsule, wine, tea and other health products [3]. Modern research has demonstrated that the polysaccharides, echinacoside, verbascoside, geniposidic acid and catalpol in Cistanche have inestimable hypoglycaemic [4], anti-inflammatory [5] and anti-Alzheimer’s disease effects and protect various organ systems in the body [6]. However, fresh Cistanche samples have high moisture contents and tend to spoil during the harvest, transport and storage processes. Therefore, Cistanches generally need to be dried to extend their storage time and improve their economic benefits.

Far-infrared vacuum drying is a new drying technology that uses infrared radiation to heat materials in a vacuum environment. Compared with hot air drying and freeze-drying, far-infrared vacuum drying has a high drying efficiency and good heating uniformity [7,8]. Far-infrared vacuum drying has been utilized to process agricultural products such as pumpkin, wolfberry and lemon [9,10,11]. However, the drying efficiency of far-infrared vacuum drying technology is greatly restricted by the moisture state and material contents, which further affects the active ingredient content in the dried products. Therefore, selecting a suitable pre-treatment method is crucial for retaining the active ingredients in Cistanche and improving the quality of its dried products.

Studies have shown that compared to methods such as blanching, ultrasound (US) can weaken the force between the moisture and materials and reduce the resistance of moisture migration through its cavitation and mechanical effect. Research results on nectarines, carrots and apples [12,13,14] showed that the drying rate was significantly increased after an US treatment [15]. In addition, studies by Villamiel et al. [16] on strawberries and carrots and by Dehsheikh et al. [17] on banana slices found that US treatments can inhibit the enzymatic activity of the material and retain the maximum amount of active ingredients in the material.

In the present study, US pre-treatment technology was applied to Cistanche slices to explore the effects, such as the treatment time, frequency and power, of US pre-treatment on far-infrared vacuum drying rate and moisture ratio of Cistanche slices. The changes of phenylethanol glycoside, iridoid, polysaccharide, total phenolic and total flavonoid content, rehydration rate and colour of the dried products were analysed to provide technical support for the actual production of Cistanche.

## 2. Materials and Methods

### 2.1. Chemicals

Echinacoside (standard), rhodioloside (standard), poliumoside (standard), verbascoside (standard), isoacteoside (standard), geniposidic acid (standard), leonurusoside (standard), catalpol (standard), phenol, 1,1-diphenyl-2-trinitrophenylhydrazine (DPPH), catechin, ascorbic acid, forinol and acetonitrile (HPLC grade) were used. The rest of the reagents, such as methanol, were analytically pure. All reagents were purchased from Chengdu Pfeiffer Biotechnology Co., Ltd., Chengdu, China.

### 2.2. Plant Material and Processing

Fresh Cistanches were purchased from Minqin, Wuwei, Gansu Province, China in the spring of 2021. After being purchased, Cistanches were immediately laid out in a refrigerator and refrigerated at 2–4 °C for a week. Cistanche slices were placed in a blast drying oven at 103 °C for 72 h [18,19]. The initial moisture content (Mc) of the sample was 78.94 ± 0.5% w.b. [20]. The Cistanche samples with a uniform length and complete shape were selected, cleaned, cut into round sheet with thickness of 4 mm slices with a slicer (KD-0281, Guangdong Baiyuan Industrial Co., Ltd., Guangzhou, China), weighed (120 ± 1 g) and placed in a 300 × 240 × 150 mm ultrasonic cleaner (KQ-300 VDE, Kunshan Ultrasonic Instrument Co., Ltd., Kunshan, China). The deionized water was added to the water level of 80 mm. According to the previous exploratory experiment, the ultrasonic treatment time, ultrasonic frequency and ultrasonic power were selected as the test indexes for the single factor experiment. The specific drying conditions are shown in Table 1. During the pretreatment process, the temperature of deionized water in the temperature ultrasonic instrument will gradually increase. The longer the ultrasonic time is, the more the temperature will increase. After 45 min of ultrasonic treatment, the temperature is 45 °C.

After an US treatment, the surface moisture of the samples was dried, and the samples were spread on a screen and placed in a far-infrared vacuum drying oven (WSH-60-A, Tianshui Shenghua Microwave Technology Co., Ltd., Tianshui, China). Through the preliminary experiment, the drying temperature of the drying oven was set as 55 °C, the irradiation height was 310 mm, and the vacuum degree was −60 kPa. The weighing system of the drying box is connected with the control system. The temperature, material mass and vacuum in the drying box are monitored in real time through the screen of the control system. The water content of the material is calculated through the material mass and record measurements every 10 min. The test was stopped when the moisture content of the Cistanches dropped to 10% w.b. [19], and the weighing system in the oven was used to record measurements every 10 min. To ensure the test data were accurate and the accidental errors were reduced, each test was repeated three times, and the average value was taken as the test result.

### 2.3. Preparation of the Test and Reference Solutions

#### 2.3.1. Preparation of the Reference Solutions for Phenylethanoid Glycosides and Iridoids

The dry Cistanche products were crushed, sieved by 0.177 mm, weighed to 1 g (AUW-220D, Shimadzu, Tokyo, Japan), added to 50 mL of 50% methanol in a brown bottle, soaked for 30 min, weighed and ultrasonically treated (250 W, 40 kHz) for 40 min [19]. Then, 50% methanol was added to augment the weight, and the mixture was shaken well and filtered. The filtrate was filtered through a 0.22 μm microporous membrane. Each experiment was repeated 3 times.

#### 2.3.2. Preparation of Control Solutions for Phenylethanol Glycoside and Iridoid Components 

The appropriate amounts of verbascoside, leonurusoside, echinacoside, catalpol, geniposidic acid, isoacteoside and rhodioloside controls were weighed in a 10 mL measuring flask. Then, the volume was fixed with 50% methanol solution to produce the control solution with concentrations of 0.986, 0.527, 1.00, 0.797, 0.947, 0.3, and 1.13 mg/mL, respectively. An appropriate amount of the above reference solution was added to 50% methanol and shaken well to prepare a mixed reference solution. Each experiment was repeated 3 times [19].

#### 2.3.3. Preparation of Polysaccharide, Total Phenol, Total Flavonoid and Antioxidant Test Solutions 

The dried Cistanches were crushed, sieved by 0.177 mm, weighed accurately to 0.5 g, added to 20 mL of 70% ethanol in a plugged triangle bottle and placed in a shaker (TS-200 B, Shanghai Jinwen Instrument Equipment Co., Ltd., Shanghai, China) (180 r/min) for 48 h under dark conditions at room temperature. Centrifugation (TG22-WS, Changsha Xiangrui Centrifuge Co., Ltd., Changsha, China) for 10 min at 4 °C and 5000 r/min was performed. The extraction of supernatant 18 mL, with 70% ethanol volume to 25 mL and was stored in a refrigerator at 4 °C for one week. An ultraviolet–visible spectrophotometer (V-5100, Shanghai Yuanxi Instrument Co., Ltd., Shanghai, China) was used to determine the polysaccharide, total phenol, and total flavonoid contents and the antioxidant capacity. Each experiment was repeated 3 times [21].

### 2.4. Calculation of Drying Parameters

#### 2.4.1. Calculation of the Dry Base Moisture Content

The wet base moisture content was calculated [22]:

(1) $$W_{t}=\frac{M_{t}-M_{d}}{M_{t}}$$

where *W_t_* represents the dry basis moisture content of the Cistanches at *t* time, %; *M_t_* represents the weight of Cistanches sliced at *t* time, g; and *M_d_* denotes the weight of dry matter, g.

#### 2.4.2. Calculation of the Moisture Ratio

The moisture ratio of the Cistanche slices was calculated [23]:

(2) $$MR=\frac{W_{t}-M_{e}}{M_{c}-M_{e}}$$

where *M_c_* represents the initial moisture content of the Cistanche, %; *M_e_* represents the equilibrium moisture content of the Cistanche, %.

The equilibrium moisture content of Cistanche *M_e_* is smaller than that of *W_t_* and *M_c_* because the equilibrium moisture content indicates that the material loses water and absorbs water to reach dynamic equilibrium. When calculating the water ratio, the equilibrium moisture content can be regarded as 0, so the calculation formula of water ratio can be simplified. The simplified moisture ratio is calculated according to Afzal and Abe [23] using Equation (3).

(3) $$MR=\frac{W_{t}}{M_{c}}$$

#### 2.4.3. Calculation of the Drying Rate

The drying rate of the Cistanche slices was calculated using Equation (4) [24].

(4) $$DR=\frac{\Delta M}{\Delta t}$$

where Δ*M* represents the water loss of the Cistanche slices at two drying times, g, and Δ*t* represents the time interval of two drying times, min.

### 2.5. Calculation of the Effective Moisture Diffusivity (D_eff_)

Effective moisture diffusion coefficient is one of the essential parameters to calculate and simulate the moisture migration mechanism of drying materials. According to the previous exploratory experiments on Cistanche slices, it was found that the drying rate of the material was mainly at the deceleration stage. Therefore, Fick’ s second law was used to describe the water diffusion process inside the material. It is calculated by Equation (5) [25].

(5) $$\ln MR=\ln\left(\frac{8}{\pi^{2}}\right)-\left(\frac{\pi^{2}D_{eff}}{4L_{0}^{2}}t\right)$$

where *MR* represents the moisture ratio of Cistanche slices; *D_eff_* represents the effective moisture diffusion coefficient of Cistanche slices, m/s^2^; *t* represents the drying time, s; and *L*_0_ represents the 1/2 slice thickness of Cistanche, mm.

### 2.6. Determination of the Quality of Dry Products 

#### 2.6.1. Determination of Phenylethanoid Glycosides and Iridoid

The contents of phenylethanoid glycosides and iridoids in Cistanche were determined by HPLC. The chromatographic conditions were as follows: Agilent Eclipes XDB-B-C-18 (250 mm × 4.6 mm, 5 μm) column, column temperature of 30 °C, volume flow rate of 1.0 mL/min, gradient elution mobile phase of acetonitrile (A)-water (B); 0~8 min, 2~5% A; 8~22 min, 5~15% A; 22~40 min, 15~35% A; 40~45 min, 35~65% A; and detection wavelength of 191 mm. Each experiment was repeated 3 times [26].

#### 2.6.2. Determination of the Polysaccharide, Total Phenol, Total Flavonoid and Antioxidant Capacity of the Dry Products

- Determination of the polysaccharide content

Referring to the method of Dubois et al. [27], the polysaccharide content of Cistanche was determined by the phenol sulfate method. Sucrose was used as the standard for calibration, and each experiment was repeated 3 times. The content of polysaccharide and the equation of the standard curve are shown in Equations (6) and (7).

(6) $$\mathrm{Content}=\frac{C_{s}\times V_{s2}}{V_{s1}\times M_{s}}$$

(7) $$Y=0.012x+0.886\;R^{2}=0.995$$

where *C_s_* represents the concentration of polysaccharide, mg/mL; *V_s_*_1_ represents the volume of the sample solution in mL; *V_s_*_2_ represents the volume of the extract in mL; and *M_s_* represents the dry weight of Cistanche, g.

- Determination of total phenolic content

Referring to the method of Beato et al. [28], the total phenolic content of Cistanche was determined by the Folin-Ciocalteu reagent. Gallic acid (GAE) was used as the standard for calibration, and each experiment was repeated 3 times. The content of total phenolic compounds and the equation of the standard curve are shown in Equations (8) and (9).

(8) $$\mathrm{Content}=\frac{C_{p}\times V_{P2}}{V_{p_{1}}\times M_{p}}$$

(9) $$Y=0.036x+0.032\;R^{2}=0.997$$

where *C_p_* represents the concentration of gallic acid in mg/mL; *V_p_*_1_ represents the volume of the sample solution in mL; *V_p_*_2_ represents the volume of the extract in mL; and *M_p_* represents the dry weight of Cistanche, g.

- Determination of antioxidant capacity

Referring to the method of Nencini et al. [29], the antioxidant capacity of Cistanche was determined by the DPPH method. Each experiment was repeated 3 times. The antioxidant capacity was calculated as Equation (10):

(10) $$\mathrm{Rate}=\frac{A_{0}-A}{A_{0}}\times 100\%$$

where *A* denotes the absorbance of the sample solution, and *A*_0_ denotes the absorbance without the sample solution.

- Determination of the total flavonoid content

Referring to the method of Lay et al. [30], the total flavonoid content of Cistanche was determined by the sodium nitrite–aluminium nitrate-sodium hydroxide method. Catechin (CE) was used as the standard for calibration, and each experiment was repeated 3 times. The total flavonoid content and the equation of the standard curve are shown in Equations (11) and (12).

(11) $$\mathrm{Content}=\frac{C_{f}\times V_{f2}}{V_{f1}\times M_{f}}$$

(12) $$Y=0.005x+0.031\;R^{2}=0.995$$

where *C_f_* denotes the concentration of catechin in mg/mL; *V_f_*_1_ represents the volume of the sample solution aspirated in mL; *V_f_*_2_ represents the volume of the extract in mL; and *M_f_* represents the weight of dry Cistanche matter, g.

#### 2.6.3. Determination of Colour Differences

The colour of dried Cistanche slices reflects the drying quality. The L*, a* and b* of fresh Cistanche slices and their dry products were measured with a colorimeter (CR-10 colorimeter, Konica Minolta Ltd., Tokyo, Japan) under natural light for three times in ach group, and the average value was taken as the experimental data. Δ*E* was calculated [31]:

(13) $$\Delta E=\sqrt{{\left(\Delta L^{*}\right)}^{2}+{\left(\Delta a^{*}\right)}^{2}+{\left(\Delta b^{*}\right)}^{2}}$$

Note: ΔL* = L-L*; Δ*a* = a-a*; Δ*b* = b-b*, where ∆*E* represents the colour difference of dried Cistanche slices; L*, a*, b* represent the brightness, red-greenness and yellow-blueness of the dried Cistanche products, and L, a, b denote the brightness, red-greenness and yellow-blueness of fresh Cistanche slices.

#### 2.6.4. Calculation of the Rehydration Rate

Rehydration is commonly used to evaluate the degree of damage in the organizational structure during the process of drying materials [32]. The larger the rehydration rate is, the smaller the degree of material structure damage, and the better the drying effect. The dried slices of Cistanche (3 g) were immersed in 300 mL of distilled water at 30 °C for 60, 120, 180, 240, 300 and 360 min. The samples were dried with filter paper and weighted on a digital balance with accuracy of ±0.001 g (AUW-220D, Shimadzu, Tokyo, Japan) [33]. Each experiment was repeated 3 times. The rehydration rate is calculated:

(14) $$R_{f}=\frac{G_{f}}{G_{g}}$$

where *R_f_* denotes the rehydration rate; *G_f_* represents the mass of Cistanche slices after rehydration, g; and *G_g_* represents the mass of Cistanche slices before rehydration, g.

#### 2.6.5. Microstructure

The sample to be tested was fixed in 2.5% glutaraldehyde iso-osmotic solution for 12 h, rinsed with 0.1 mol/L, pH 7.2 phosphate buffer solution three times (5–15 min each time), and dehydrated with 50%, 70%, 80%, 90%, 100% ethanol in turn (15 min each time). The dehydrated materials were dried overnight in tert-butyl alcohol solution at room temperature, sprayed with gold for 60 s, and amplified by scanning electron microscopy (S-4800N, Hitachi Corporation, Tokyo, Japan) 500 times to observe the microstructure of the sample surface, and the electron microscopic parameters of each sample were marked in the lower left corner of Figure 6.

### 2.7. Statistical Analysis

The significance of the effects of US treatment time, US frequency and US power on the drying rate, active ingredient content and rehydration rate were determined by conducting one-way analyses of variance (ANOVA). Duncan’s multiple comparison tests was performed using SPSS 22.0 (SPSS IBM Corporation, Armonk, NY, USA). Statistical significance for differences was tested at the 5% probability level (*p* < 0.05). The optimal test conditions are evaluated by entropy method.

## 3. Results and Discussion

### 3.1. Effect of US Pre-Treatment on the Process of Drying Cistanche Slices by Far-Infrared Vacuum Drying

#### 3.1.1. US Treatment Time

**Figure 1:**
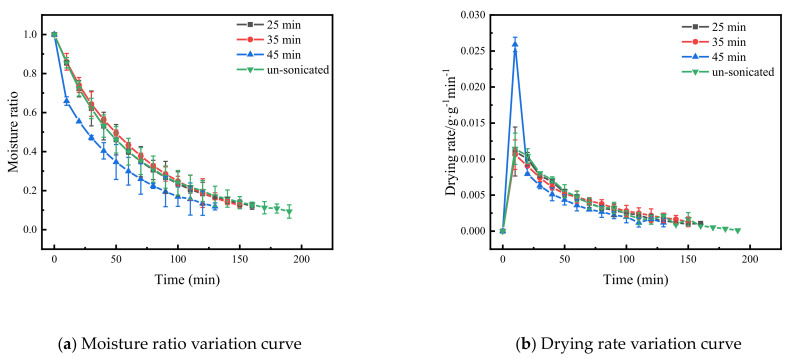
The characteristic curve of far-infrared vacuum drying of Cistanche slices under different ultrasonic treatment times. The vertical bars indicate the standard deviation from the mean.

Based on pre-experiment, when the US frequency was 40 kHz, the power was 180 W and the processing time was 0, 25, 35 and 45 min; the drying characteristic curve of Cistanche slices is shown in Figure 1. With increasing US treatment time, the time to reach safe moisture content (10% w.b.) in the Cistanche slices gradually decreased. Compared with the samples without the US treatment, the drying time of the material was reduced by 21%, 31% and 31.5% after the US treatment was applied for 25 min, 35 min and 45 min, respectively. This is because the water molecules are continuously stretched and compressed by US, which weakens the force between the material components and reduces the resistance to moisture migration [34]. In addition, the periodic compression and expansion of US waves increases the pore space between the Cistanches cells, accelerating the convective mass transfer rate of materials and enhancing the drying efficiency of materials. In addition, it was evident that the drying rate of Cistanche slices gradually decreased in the middle and late stages of drying, which was related to the internal moisture content of the material. A similar trend was found by Luangmalawat et al. [35] when drying rice. As the difference in drying time of Cistanche slices was small between the US treatments of 35 min and 45 min, it is recommended to set the US pre-treatment time range to 25–35 min in order to save resources and reduce energy consumption.

#### 3.1.2. US Frequency

**Figure 2:**
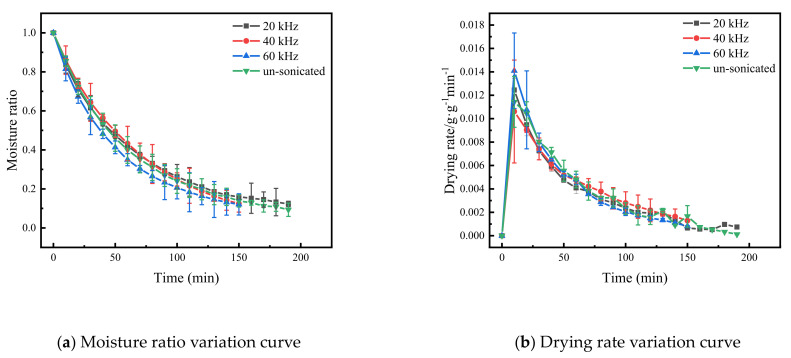
The characteristic curve of far-infrared vacuum drying of Cistanche slices under different ultrasonic frequencies. The vertical bars indicate the standard deviation from the mean.

Based on pre-experiment, when the US treatment time was 35 min, the US power was 180 W and the US frequency was 0, 20, 40 and 60 kHz, and the drying characteristic curve of the Cistanche shown in Figure 2. The curves showing the moisture ratio change and drying rate change in Cistanche slices almost coincide. This indicates that the US frequency has little effect on the process of drying Cistanche slices with far-infrared vacuum drying. However, under the same drying conditions, compared with the samples without ultrasonic treatment and the samples with ultrasonic frequency of 20 kHz, the drying time of the samples with ultrasonic frequency of 40 kHz and 60 kHz was shortened by 16% (*p* < 0.05) which may be because the ultrasonic frequency was small enough to support the formation of new microscopic channels in Cistanche. After increasing the frequency, the effect of ultrasound was enhanced, and the migration rate of water in the drying process was accelerated [36,37].

#### 3.1.3. US Power

**Figure 3:**
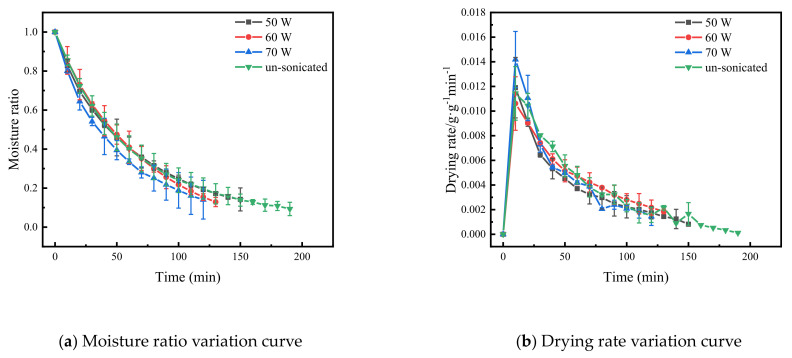
The characteristic curve of far-infrared vacuum drying of Cistanche slices under different ultrasonic power. The vertical bars indicate the standard deviation from the mean.

Based on the pre-experiment, when the US treatment time was 35 min, the frequency was 40 kHz and the US power was 0, 150, 180 and 210 W, and the drying characteristic curve of the Cistanche slices is shown in Figure 3. Compared to the US treatment time and US frequency, the effect of US power on the drying rate of the materials was more significant. The stronger the US power was, the more obvious the improvement in the drying rate of the Cistanche slices. Compared to the samples without sonication, the drying time of the Cistanche slices was reduced by 21%, 31.5% and 36.8% at US 150, 180 and 210 W, respectively. The drying pattern was similar to that of purple potato [38] and apple [14]. This may be because high-frequency mechanical oscillation exerts a crushing and tensional effect on the ultrasonic medium, and this effect breaks the bonds between macromolecules, such as proteins and polysaccharides, and water molecules in the material [39]. The rate of water removal from the surface of the material was accelerated by the conversion of water that was bound and difficult to remove to water that was free and more mobile [40]. In addition, the cavitation and mechanical effects of US generate many tiny vacuoles around the material. The strong shear stress caused by the rupture of the vacuoles cause the material to become loose and internally porous, reducing the resistance to water migration [36].

### 3.2. Effective Moisture Diffusivity

The effective moisture diffusivity of far-infrared vacuum drying of Cistanche slices under different US conditions presented in Table 2. The effective moisture diffusivity coefficient of Cistanche slices ranged from 1.611 22 × 10^−8^ to 2.392 74 × 10^−8^ m^2^/s, which conforms to the range of food processing (10^−11^~10^−8^) [41]. The effective moisture diffusivity coefficient of Cistanche slices increased correspondingly with the increase in US treatment time, power and frequency. This may be due to the increase in internal pore channels, mass transfer rate, drying rate and amount of water diffusion per unit area after the US treatment. This suggests that the internal structure and drying rate of Cistanches are the key factors that affect their effective moisture diffusion coefficient. This result is consistent with the findings of mushrooms and blueberries [42,43].

### 3.3. Effect of US Pre-Treatment on the Drying Quality of Cistanche

#### 3.3.1. Phenylethanol Glycosides and Iridoids

Phenylethanol glycosides and iridoids are the main compounds of Cistanche with anti-inflammatory and analgesic effects. The contents of phenylethanol glycosides and iridoids in Cistanche slices under different US conditions are shown in Table 3. Compared to the unsonicated samples, the retention of phenylethanol glycosides and iridoids in the material after the US treatment was significantly increased. This result indicated that the US pre-treatment was beneficial in reducing the loss of active ingredients in the material. At the same time, the degree of influence of different US conditions on each phenylethanol glycoside and iridoid component varied greatly. For example, when the US frequency was increased from 40 to 60 kHz, the content of rhodioloside decreased by 84%, but there was no significant difference in the echinacoside, poliumoside, verbascoside and isoacteoside contents. The US frequency increased from 180 to 210 W, and the echinacoside, rhodioloside, poliumoside, verbascoside, isoacteoside and geniposidic acid contents increased by 23%, 43%, 15%, 14%, 32% and 13%, respectively. However, there was a 39% and 13% decrease in the content of leonurusoside and catalpol, respectively. This may be due to the poor stability of leonurusoside and catalpol, as their molecular structures were damaged by the transient high temperature that was generated by the US [44]. In addition, when the US treatment time exceeded 35 min, the temperature of the US medium increased, and the phenylethanol glycoside and iridoid contents gradually decreased.

#### 3.3.2. Polysaccharides

**Figure 4:**
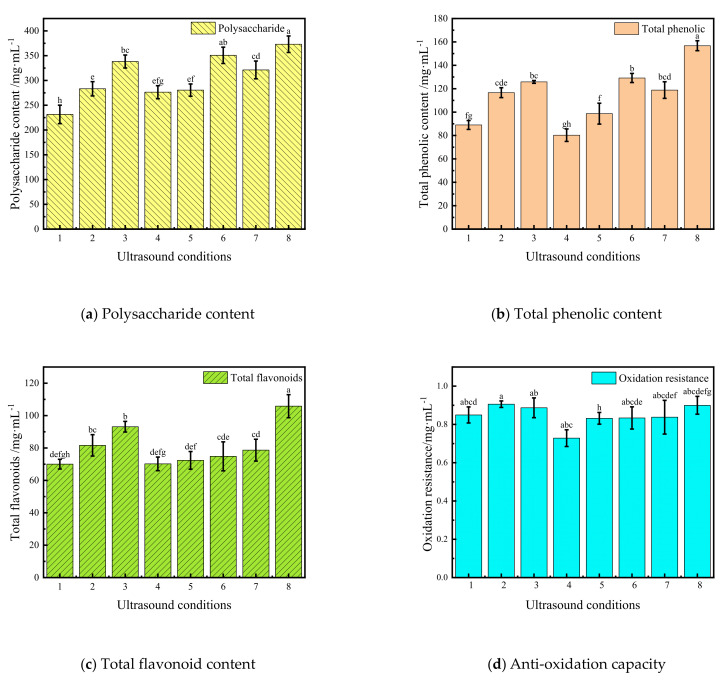
Effects of different ultrasonic conditions on polysaccharides, total phenols, total flavonoids and antioxidant capacity of Cistanche. Ultrasound conditions: 1 (UN-sonicated), 2 (US 25 min, 40 kHz, 180 W), 3 (US 35 min, 40 kHz, 180 W), 4 (US 45 min, 40 kHz, 180 W), 5 (US 35 min, 20 kHz, 180 W), 6 (US 35 min, 60 kHz, 180 W), 7 (US 35 min, 40 kHz, 150 W), 8 (US 35 min, 40 kHz, 210 W). The vertical bars indicate the standard deviation from the mean. The letters reveal significant differences (*p* < 0.05) according to the Duncan test.

Studies have shown that the polysaccharides in Cistanche have immune enhancing, anti-tumour and anti-inflammatory effects [45]. The variation pattern of polysaccharide content in Cistanche slices under different sonication conditions is shown in Figure 4a. When the US frequencies were 20, 40 and 60 kHz, the polysaccharide content increased by 21%, 46% and 51%, respectively, compared with the samples without sonication. At US powers of 150, 180 and 210 W, the polysaccharide content increased by 38%, 46% and 61%, respectively, under the same drying conditions. Fei et al. [46] also obtained similar trends in a study on mushrooms. Reducing the time that the material was exposed to high temperatures in the far-infrared vacuum drying oven after the US pre-treatment could reduce the Maillard reaction and enzymatic activity of polysaccharides. Therefore, increasing the US frequency or power under these conditions can reduce the loss of polysaccharide content in the dried product. However, if the parameter value is too large, the quality of dry products will decrease significantly. For example, when the US treatment time was increased from 35 min to 45 min, the polysaccharide content of the material decreased by 18%. This result indicated that the polysaccharide content may also be limited by other conditions and that drying time is not the only influencing factor.

#### 3.3.3. Total Phenolic Content

Phenols have received much attention for their strong anti-inflammatory and antibacterial properties [47]. The variation pattern of the total phenol content in Cistanche slices under different sonication conditions is shown in Figure 4b. The total phenol content increased by 40% after 35 min of US pre-treatment compared to the sample without ultrasound treatment. The total phenol content increased by 45% when the ultrasound frequency was 60 kHz and by 86% when the ultrasound power was 210 W. It was similar to the results obtained by Ren et al. [48] with onions [49]. This may be because the bond connecting the phenolic compounds and the macromolecules in the material ruptures under the action of US. The release of polyphenols increased the total phenolic content [50]. In addition, the content of total phenols in Cistanches is closely related to the heating temperature and heating time. Consistent with Kahraman et al. research on apple [51]. As shown in Figure 1, Figure 2 and Figure 3, the longer the US treatment time is, the higher the US power and frequency, the shorter the drying time required for the material, the lower the probability of oxidation, polymerization or decomposition of phenolic compounds, and the higher the total phenolic content. However, when the US treatment time was 45 min, the content of total phenols in Cistanches was only 80.29 mg, which was significantly lower than that under the other conditions (*p* < 0.05) [52]. This may be the creation of reactive forms of oxygen during US treatment, which oxidises the phenolics [36]. Therefore, to ensure the quality of dried Cistanche products, the US treatment time should be less than 45 min.

#### 3.3.4. Total Flavonoids

The flavonoid content in Cistanche is high and has various effects, such as antithrombotic and hypolipidaemic effects [53]. Figure 4c reflects the variation pattern of the total flavonoid content in Cistanche slices under different US conditions. The effects of various ultrasonic factors on the total flavonoid content were statistically significant (*p* < 0.05). The content of flavonoids in the materials after US treatment was higher than the 70.01 mg observed in the untreated samples. In addition, the effect of US power on flavonoids was more significant than that of US treatment time and US frequency. There was a 34% increase in total flavonoid content at 210 W compared to 150 W. Rodriguez et al. [52] found that ultrasonic combined with low-temperature drying could significantly reduce the loss of polyphenols and flavonoids and maintain high antioxidant activity compared with ordinary thermal drying. This could be because the higher the power is, the stronger the influence of US is on the material. The strong shear force generated by the cavitation effect damaged the Cistanche cell walls, which promoted the release of intracellular flavonoid substances and increased the content of active ingredients in the material. However, as flavonoids are thermally sensitive components, their stability is poor and easily affected by the temperature of the US medium [54,55].

#### 3.3.5. Anti-Oxidation Capacity

DPPH is commonly used to evaluate the ability of antioxidants to scavenge free radicals [56]. The effect of different drying conditions on the antioxidant capacity of Cistanche is shown in Figure 4d. The antioxidant activity of Cistanche was optimal at a US treatment time of 35 min, a US frequency of 40 kHz and a US power of 210 W. In addition, the trend of the antioxidant capacity of Cistanche under different US conditions was the same as that of the total phenol and total flavonoid. Thus, the antioxidant capacity of Cistanche may be influenced by the total phenol and total flavonoid contents. This view is generally consistent with the findings of Fu et al. [57]. However, the difference in the antioxidant capacity of the materials under different conditions was less significant (*p* < 0.05). This might be due to the metabolites of some monophenols and flavonoids in the materials after the free radical scavenging capacity of Cistanche is improved after the US treatment [58].

### 3.4. Colour

The colour of the material is related to oxidation, enzymatic browning and the Maillard reaction [59]. As the samples were dried in a vacuum environment, there was less browning due to oxidation [60]. The pattern of changes in colour parameters of Cistanche slices under different US conditions can be seen in Table 4. The colour differences of Cistanche slices after sonication were all lower than those of the untreated dried product, which could be attributed to the increased drying rate of the material reducing the probability of the Maillard reaction [61]. In addition, the colour difference of the dried products after US pre-treatment for 45 min was reduced by 40% compared to that of the US pre-treatment for 25 min. This may be because the US treatment time is too long, the material cells rupture, and the intracellular oxidase flows out. This affects the production of melanin-like substances after quinone polymerization, which increases the ΔL* value and decreases the Δa* and Δb* values. A similar trend was found in the study by Ahmed et al. [62] with papaya puree.

### 3.5. Rehydration Rate

**Figure 5:**
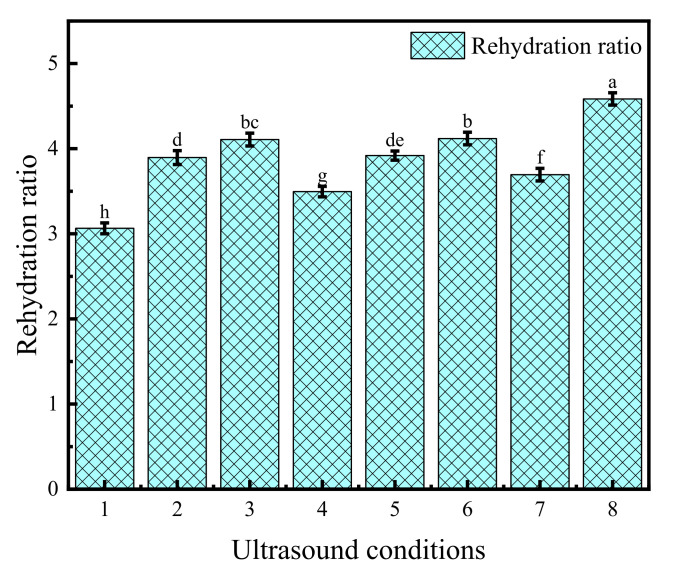
Rehydration rate of Cistanche slices under different ultrasonic conditions. Ultrasound conditions: 1 (UN-sonicated), 2 (US 25 min, 40 kHz, 180 W), 3 (US 35 min, 40 kHz, 180 W), 4 (US 45 min, 40 kHz, 180 W), 5 (US 35 min, 20 kHz, 180 W), 6 (US 35 min, 60 kHz, 180 W), 7 (US 35 min, 40 kHz, 150 W) and 8 (US 35 min, 40 kHz, 210 W). The vertical bars indicate the standard deviation from the mean. The letters reveal significant differences (*p* < 0.05) according to the Duncan test.

The variation pattern of the rehydration rate of Cistanche slices under different US conditions is shown in Figure 5. Under the same drying conditions, the rehydration rate of the dried products from US pre-treatment was stronger, which is similar to the research results of Mothibe et al. [61] on apples. The rehydration rate of Cistanche slices was the best (4.58) when the US treatment time was 35 min, the frequency was 40 kHz and the power was 210 W. This is because the high frequency oscillation and expansion effect of US expands the internal structure of the material. It reduces the resistance of water as it enters the material and improves the rehydration effect of the samples [63]. In addition, when the US pre-treatment time was 25 min, 35 min and 45 min, the rehydration rates of the materials were 3.89, 4.10 and 3.49, respectively, which is an increasing and then decreasing trend. This is related to the fact that US can promote the formation of new microporous channels inside the materials and improve the rehydration rate of the dry products [64]. However, an excessively long US treatment time will destroy the organizational structure of the materials and make the surface cells lose their vitality, resulting in reduced rehydration rate.

### 3.6. Microstructure

**Figure 6:**
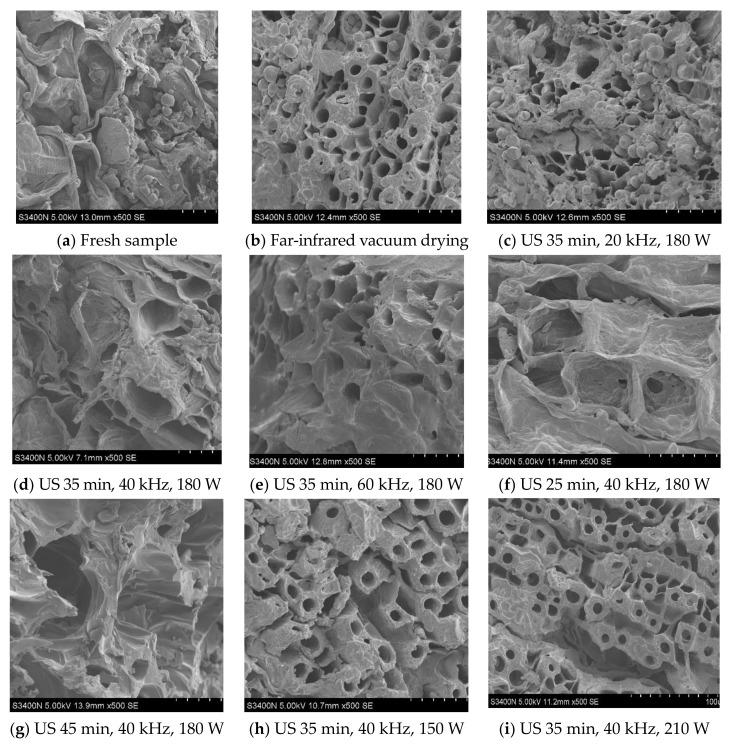
Scanning electron microscopic images of the surface of Cistanche slices under different ultrasonic conditions. The electron microscopic parameters of each sample were marked in the lower left corner.

The microstructures of Cistanche slices under different US conditions are shown in Figure 6. By comparing the microstructure of each sample, it was found that the fresh Cistanche slices (Figure 6a) had tightly arranged cells, high surface smoothness and a few pores. The number of pores in dried Cistanche slices increased significantly, and similar results were reported by Zhang et al. [65] in their study on strawberries. Compared with the samples with far-infrared vacuum drying only (Figure 6b), the samples with the US treatment had obvious pore size expansion, and a good honeycomb structure.

In addition, there were significant differences in the structure of the samples under different US conditions. This directly influenced the moisture migration rate and rehydration rate of the material. Compared with that observed for the US pre-treatment for 25 min (Figure 6f), after 45 min of US pre-treatment, the microstructure of the sample was destroyed in a large area and the boundary collapse was serious, which can be confirmed by the above the rehydration rate. At the same time, with increasing US frequency and power, the structure of the samples became more homogeneous and compact (Figure 6d–i). This may be because the cavitation and mechanical effects of US changed the pore structure of the material, making it fluffy and porous inside, which facilitated the evaporation of water.

## 4. Entropy

Entropy can be used as a tool in attribute evaluation [66]. Entropy method is to determine the weight according to the variation degree of each index. The greater the variation degree of entropy weight coefficient of the selected index, the higher the importance of the index. The contents of phenylethanol glycoside, iridoid, polysaccharide, total phenolic, total flavonoid content and antioxidant capacity in samples under different ultrasonic conditions and their antioxidant capacity were used as evaluation indexes. The weight of eight ultrasonic test conditions was calculated by entropy weight method. The results are as follows in Table 5. It can be found that the quality of dried products under US 45 min, 40 kHz and 180 W is the worst, and the quality of dried products under US 35 min, 40 kHz and 210 W is the best.

## 5. Conclusions

It was found that the time for the material to reach the safe moisture content (10% w.b.) was shortened by at least 16% after the US pre-treatment, and the US treatment time and US power were the main factors affecting the drying rate and the content of active components. When the US frequency is constant, increasing the US treatment time or power can reduce the drying time of the materials. The drying time was reduced by 21%, 31% and 31.5% after the US treatment was applied for 25 min, 35 min and 45 min. The drying time of the Cistanche slices was reduced by 21%, 31.5% and 36.8% at US 150, 180 and 210 W. By entropy method, it can be found that the content of active components in US 35 min, 40 kHz and 210 W is the highest. In addition, the retention of active ingredients in Cistanche is closely related to the integrity of its cell structure. After the US treatment for 45 min, the cells were severely damaged, some components were degraded under ultrasonic action, and the content of active ingredients decreased. Compared with samples without sonication, after the US pre-treatment, the overall structure was honeycomb-like, which was beneficial to the drying rate and capacity of the material to rehydrate. In conclusion, the US pre-treatment can significantly increase the drying rate of Cistanche and improve the quality of its dried products. The pre-treatment is suitable to pre-process Cistanche slices, as it has a good enhancement effect on far-infrared vacuum drying.

Future research will focus on the effects of different drying methods on the drying characteristics and drying quality of Cistanche. It is crucial to select suitable drying and pretreatment methods for the production and processing of Cistanche.

## Figures and Tables

**Table 1:** Ultrasonic single-factor test of Cistanche slices.

Experimental Number	US Treatment Time (Min)	US Frequency (kHz)	US Power (W)
1	0	0	0
2	25	40	180
3	35	40	180
4	45	40	180
5	35	20	180
6	35	60	180
7	35	40	150
8	35	40	210

**Table 2:** Effective water diffusion coefficient of Cistanche slices under different ultrasonic conditions.

US Treatment Time (Min)	US Frequency (kHz)	US Power (W)	Effective Moisture Diffusivity *D_eff_* × 10^−8^ (m^2^/s)
0	0	0	1.71886
25	40	180	1.92217
35	40	180	2.02512
45	40	180	2.39274
35	20	180	1.61122
35	60	180	2.05954
35	40	150	1.88246
35	40	210	2.36791

**Table 3:** The contents of phenylethanol glycoside and iridoid in Cistanche slices under different ultrasonic conditions.

Sample	Phenylethanol Glycoside Composition (mg)					Iridoid Composition (mg)		
	Echinacoside	Rhodioloside	Poliumoside	Verbascoside	Isoacteoside	Geniposidic Acid	Leonurusoside	Catalpol
un-sonicated	42.24 ± 4.74 ^gh^	3.33 ± 0.20 ^d^	494.64 ± 6.45 ^def^	46.55 ± 0.85 ^f^	13.43 ± 0.44 ^g^	242.75 ± 6.11 ^bc^	15.40 ± 0.77 ^a^	21.40 ± 0.77 ^h^
US 25 min, 40 kHz, 180 W	59.28 ± 3.88 ^bcd^	2.29 ± 0.23 ^ef^	510.88 ± 16.45 ^d^	48.34 ± 0.41 ^e^	26.18 ± 0.58 ^d^	231.59 ± 5.94 ^cde^	9.43 ± 0.85 ^cd^	35.51 ± 0.92 ^f^
US 35 min, 40 kHz, 180 W	64.24 ± 2.83 ^bc^	4.51 ± 0.28 ^b^	544.38 ± 14.10 ^bc^	57.28 ± 0.76 ^c^	33.32 ± 0.95 ^b^	220.60 ± 6.24 ^efg^	11.57 ± 0.35 ^b^	60.20 ± 0.77 ^b^
US 45 min, 40 kHz, 180 W	44.87 ± 3.74 ^g^	2.66 ± 0.18 ^e^	483.19 ± 15.02 ^defgh^	43.43 ± 0.38 ^h^	6.17 ± 0.44 ^h^	263.29 ± 5.63 ^a^	7.12 ± 0.45 ^g^	33.28 ± 0.90 ^g^
US 35 min, 20 kHz, 180 W	57.40 ± 5.98 ^cde^	2.20 ± 0.14 ^fg^	494.45 ± 14.55 ^defg^	46.12 ± 0.29 ^fg^	15.29 ± 0.89 ^f^	224.36 ± 6.91 ^ef^	8.55 ± 0.22 ^def^	46.59 ± 0.89 ^d^
US 35 min, 60 kHz, 180 W	67.54 ± 6.53 ^b^	0.72 ± 0.04 ^h^	556.78 ± 23.11 ^b^	60.34 ± 0.68 ^b^	32.08 ± 0.70 ^bc^	242.05 ± 9.93 ^ncd^	10.29 ± 0.30 ^c^	62.35 ± 0.92 ^a^
US 35 min, 40 kHz, 150 W	54.73 ± 2.29 ^def^	3.96 ± 0.39 ^c^	497.36 ± 13.49 ^de^	51.99 ± 0.29 ^d^	24.65 ± 0.98 ^e^	191.16 ± 6.49 ^h^	8.78 ± 0.88 ^de^	44.29 ± 0.94 ^e^
US 35 min,40 kHz, 210 W	79.63 ± 7.52 ^a^	6.48 ± 0.34 ^a^	629.66 ± 16.45 ^a^	65.34 ± 0.88 ^a^	44.07 ± 0.79 ^a^	250.38 ± 8.63 ^b^	7.02 ± 0.16 ^gh^	52.04 ± 0.97 ^c^

Note: Data are expressed as means ± standard deviation of triplicate samples. The letters in the same column reveal significant differences (*p* < 0.05) according to the Duncan test.

**Table 4:** Colour parameters of Cistanche slices under different ultrasound conditions.

Sample	L*	a*	b*	∆E*
Fresh	61.26 ± 2.15 ^a^	3.89 ± 0.46 ^h^	24.6 ± 0.64 ^h^	-
un-sonicated	46.47 ± 3.47 ^cdefgh^	10.48 ± 0.31 ^a^	31.07 ± 0.29 ^a^	17.44 ± 3.65 ^a^
US 25 min, 40 kHz, 180 W	46.97 ± 1.27 ^cde^	9.21 ± 0.76 ^b^	30.84 ± 0.35 ^ab^	16.48 ± 1.52 ^abc^
US 35 min, 40 kHz, 180 W	47.23 ± 1.58 ^cd^	8.49 ± 0.30 ^bcde^	29.86 ± 0.88 ^abcd^	15.65 ± 1.83 ^abcde^
US 45 min, 40 kHz, 180 W	52.05 ± 0.66 ^b^	5.5 ± 0.21 ^h^	27.66 ± 0.71 ^g^	9.84 ± 0.99 ^gh^
US 35 min, 20 kHz, 180 W	46.52 ± 1.93 ^cdefg^	8.54 ± 0.51 ^bcd^	29.17 ± 1.43 ^def^	16.12 ± 2.45 ^abcd^
US 35 min, 60 kHz, 180 W	46.92 ± 3.06 ^cdef^	8.37 ± 0.28 ^bcdef^	26.35 ± 0.47 ^h^	15.13 ± 3.10 ^abcdef^
US 35 min, 40 kHz, 150 W	46.34 ± 2.87 ^cdefgh^	9.17 ± 0.77 ^bc^	30.81 ± 0.95 ^abc^	16.94 ± 3.12 ^abcdef^
US 35 min, 40 kHz, 210 W	49.13 ± 2.11 ^bc^	8.31 ± 0.28 ^bcdefg^	29.28 ± 0.34 ^de^	13.73 ± 2.15 ^abcdefg^

Note: Data are expressed as means ± standard deviation of triplicate samples. The letters in the same column reveal significant differences (* *p* < 0.05) according to the Duncan test.

**Table 5:** Evaluation weight values of each ultrasonic condition.

US Treatment Time (Min)	US Frequency (kHz)	US Power (W)	Evaluation Weight	Rank
0	0	0	0.08	6
25	40	180	0.11	4
35	40	180	0.19	2
45	40	180	0.03	8
35	20	180	0.07	7
35	60	180	0.15	3
35	40	150	0.10	5
35	40	210	0.27	1

## Data Availability

The datasets generated for this study are available on request to the corresponding author.
